# Supplementary figures and images for: Single-cell and spatiotemporal profile of ovulation in the mouse ovary
Source: PLoS Biol. 2025 Jun 24;23(6):e3003193. doi: 10.1371/journal.pbio.3003193 (PMC12186953; doi:10.1371/journal.pbio.3003193)

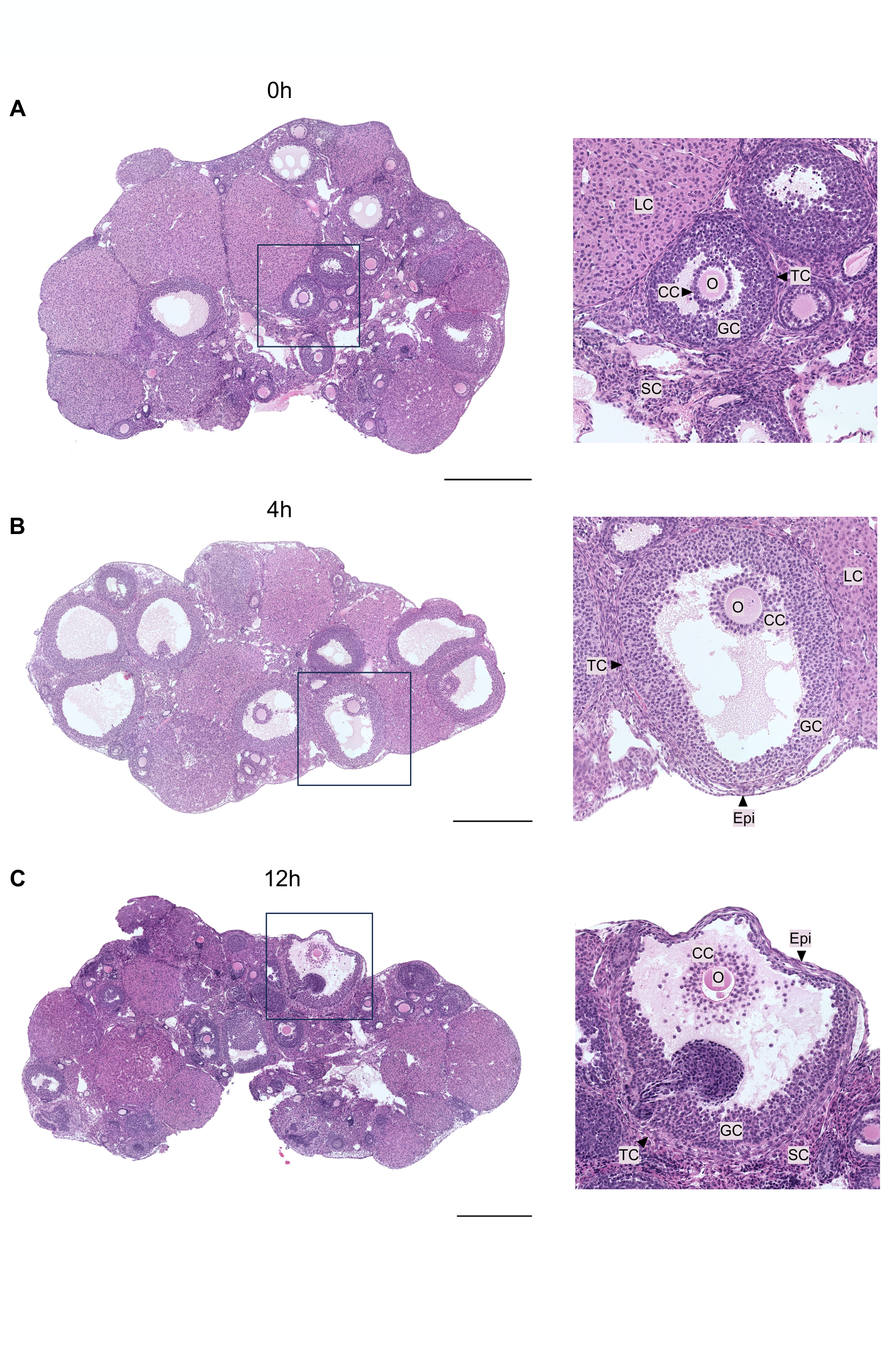

Supplement: S1 Fig — O = oocyte, CC = cumulus cells, GC = granulosa cells, TC = theca cells, SC = stroma cells, LC = luteal cells, Epi = epithelial cells. Scale bars = 200 µm. (TIFF) [file pbio.3003193.s001.tiff]

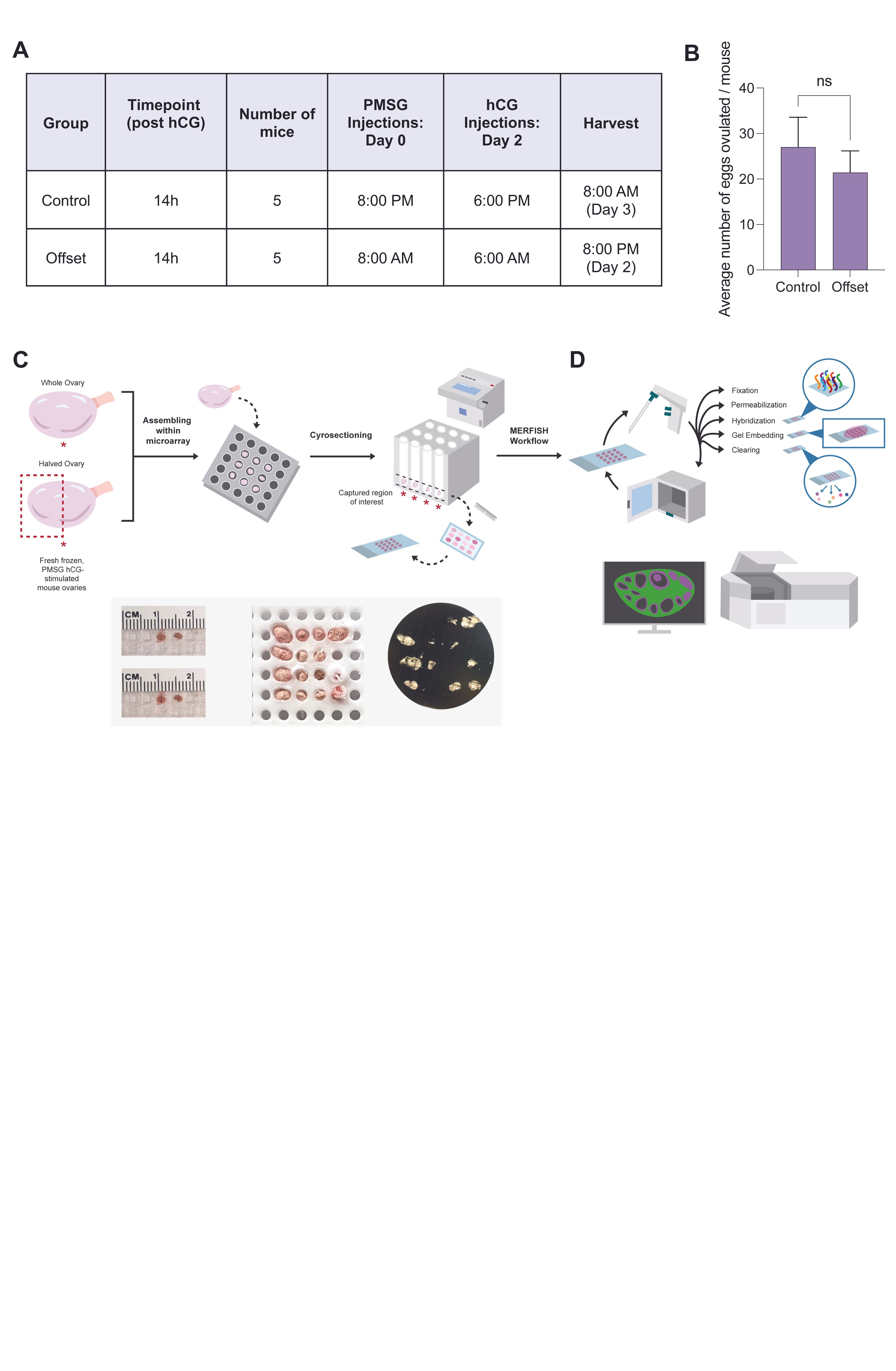

Supplement: S2 Fig — (A) Table depicting timing of hormone (PMSG and hCG) injections and ovary collection in control and offset timing groups. (B) Graph showing the average number of COCs collected per mouse in control and offset timing groups. (C) Top row: Pregnant mare serum gonadotropin (PMSG) and human chorionic gonadotropin (hCG)-stimulated, flash-frozen mouse ovaries were collected as intact whole ovaries or contralaterally-halved ovaries. Samples were embedded into a pre-formed OCT tissue microarray scaffold (TMA) with the ovarian hilums (indicated by red asterisk, *) pointing downward towards the tissue microarray base, enabling uniform tissue section collection onto fluorescent microsphere-coated functionalized coverslips. Bottom row: corresponding images of bisected ovaries, embedding into the TMA scaffold, and 10× DAPI imaging of the resulting tissue sections. (D) MERFISH protocol of mounted samples undergoing a series of staining and incubations (fixation, permeabilization, hybridization, polyacrylamide gel embedding, tissue clearing with detergents). (TIFF) [file pbio.3003193.s002.tiff]

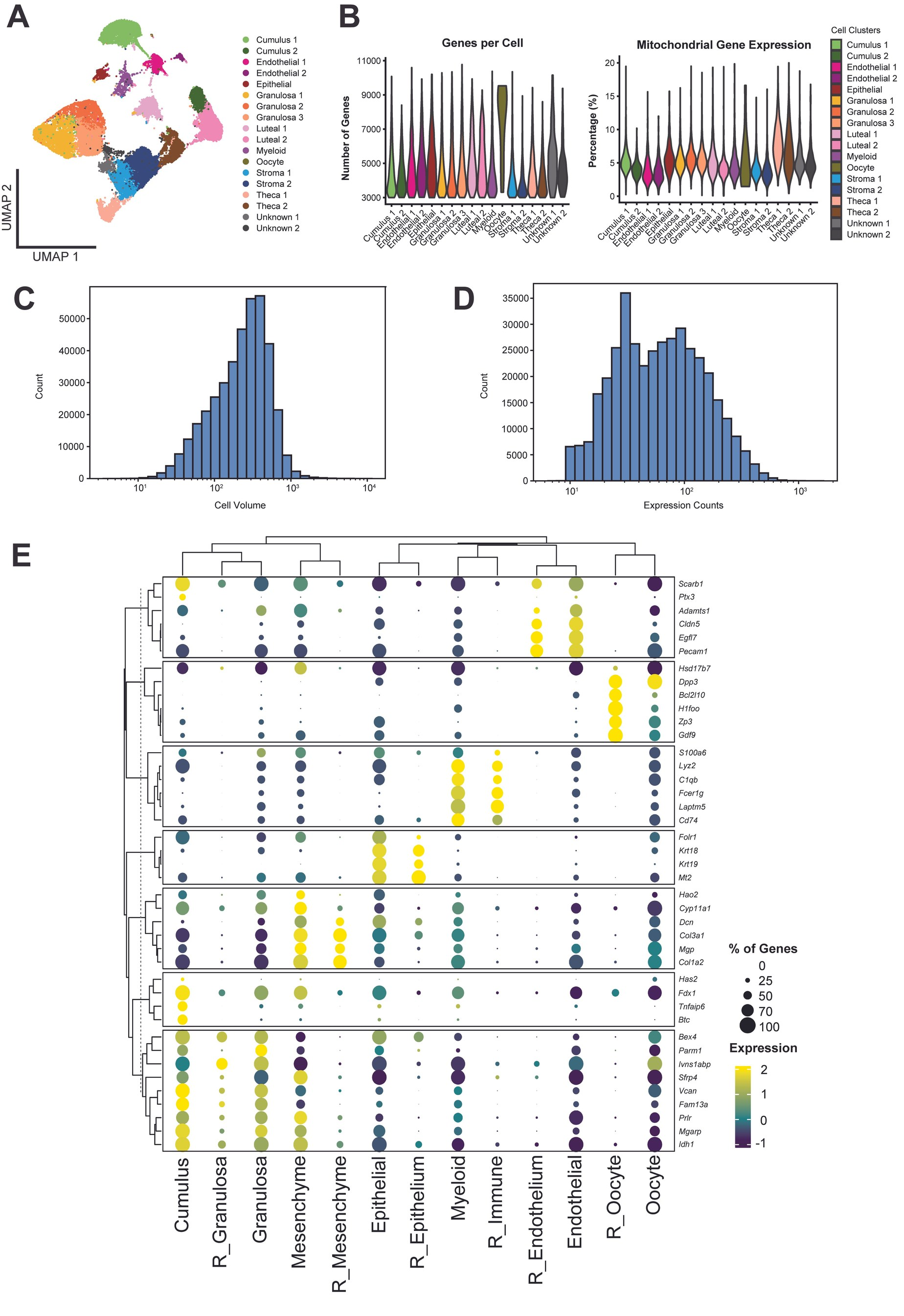

Supplement: S3 Fig — (C) UMAP of the single-cell dataset, including unknown clusters. (D) Quality metrics for single-cell dataset: Violin plots on genes per cell and percent mitochondrial in the single-cell dataset post-filtering. (E) Clustered dot plot compares cell types found in both datasets using established markers. The data underlying this figure is available at the Gene Expression Omnibus (GEO) under accession number GSE294534. (TIFF) [file pbio.3003193.s003.tiff]

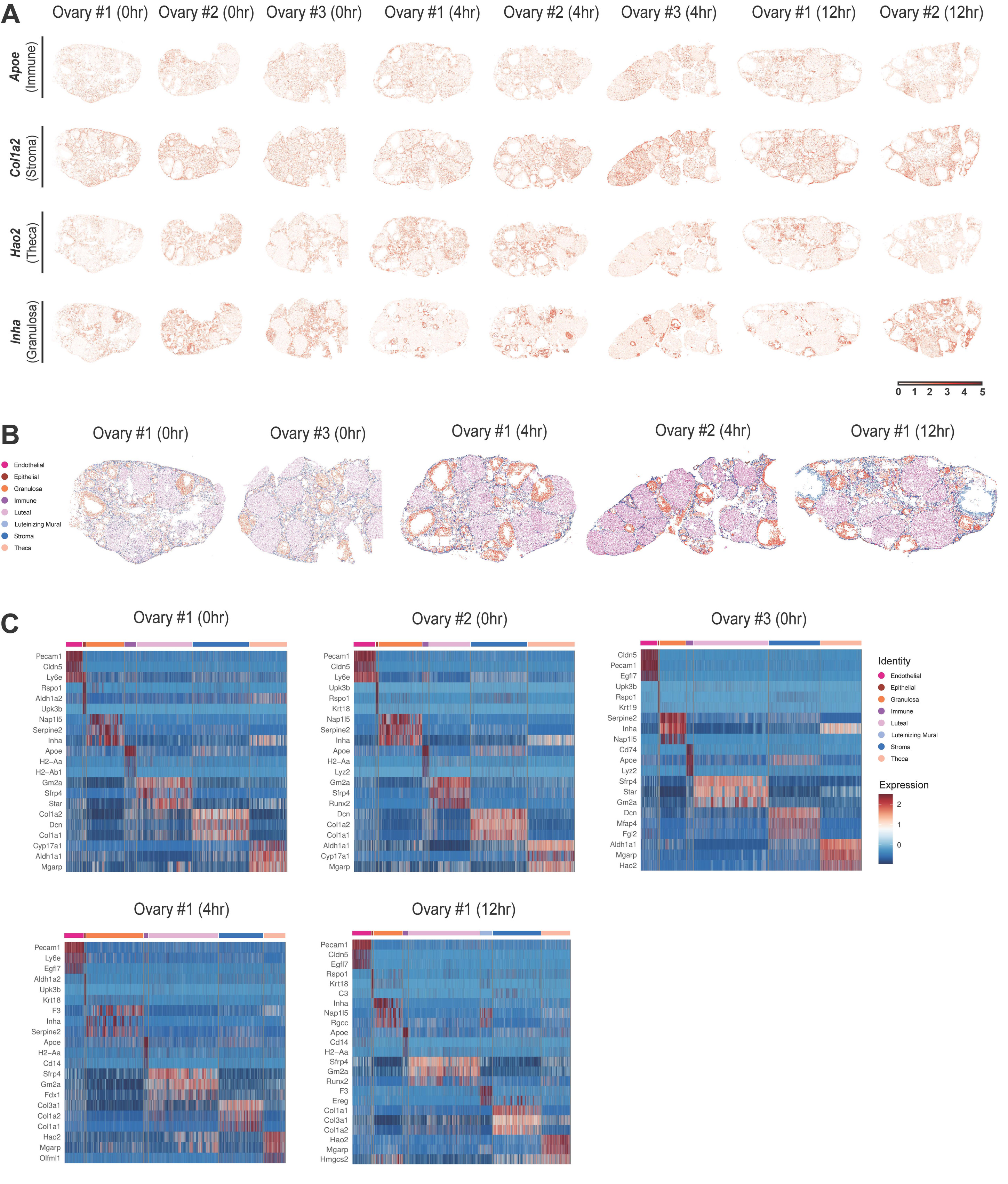

Supplement: S4 Fig — (B) Ovaries serve as biological replicates that are not included in the main figures, colored by cell types identified. (C) Heatmap showing three top marker genes used to determine the identity of each cell cluster for each ovary in iST. The data underlying this figure is available at the Gene Expression Omnibus (GEO) under accession number GSE294534. (TIFF) [file pbio.3003193.s004.tiff]

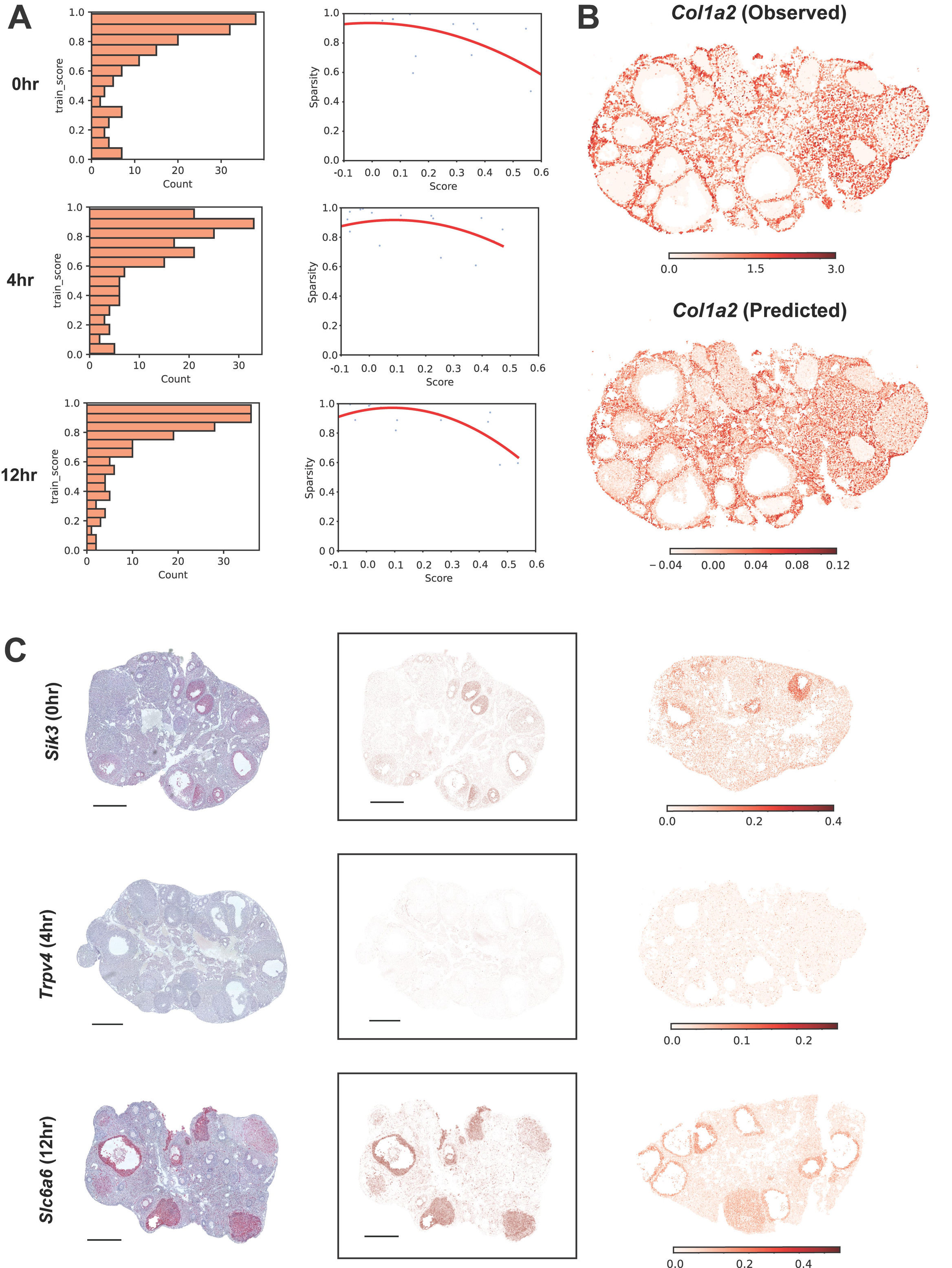

Supplement: S5 Fig — (A) Bar plots of training scores for training genes (left) and scatter plot of test scores versus sparsity for test genes (right), for the three integrations at 0 hr (top), 4 hr (middle), and 12 hr (bottom). (B) Expression plots of Col1a2 for predicted and observed results show similar patterns. (C) RNAscope images (left) and predicted results from integration analysis (right) show similar expression patterns. Scale bars = 200 µm. The data underlying this figure is available at the Gene Expression Omnibus (GEO) under accession number GSE294534. (TIFF) [file pbio.3003193.s005.tiff]

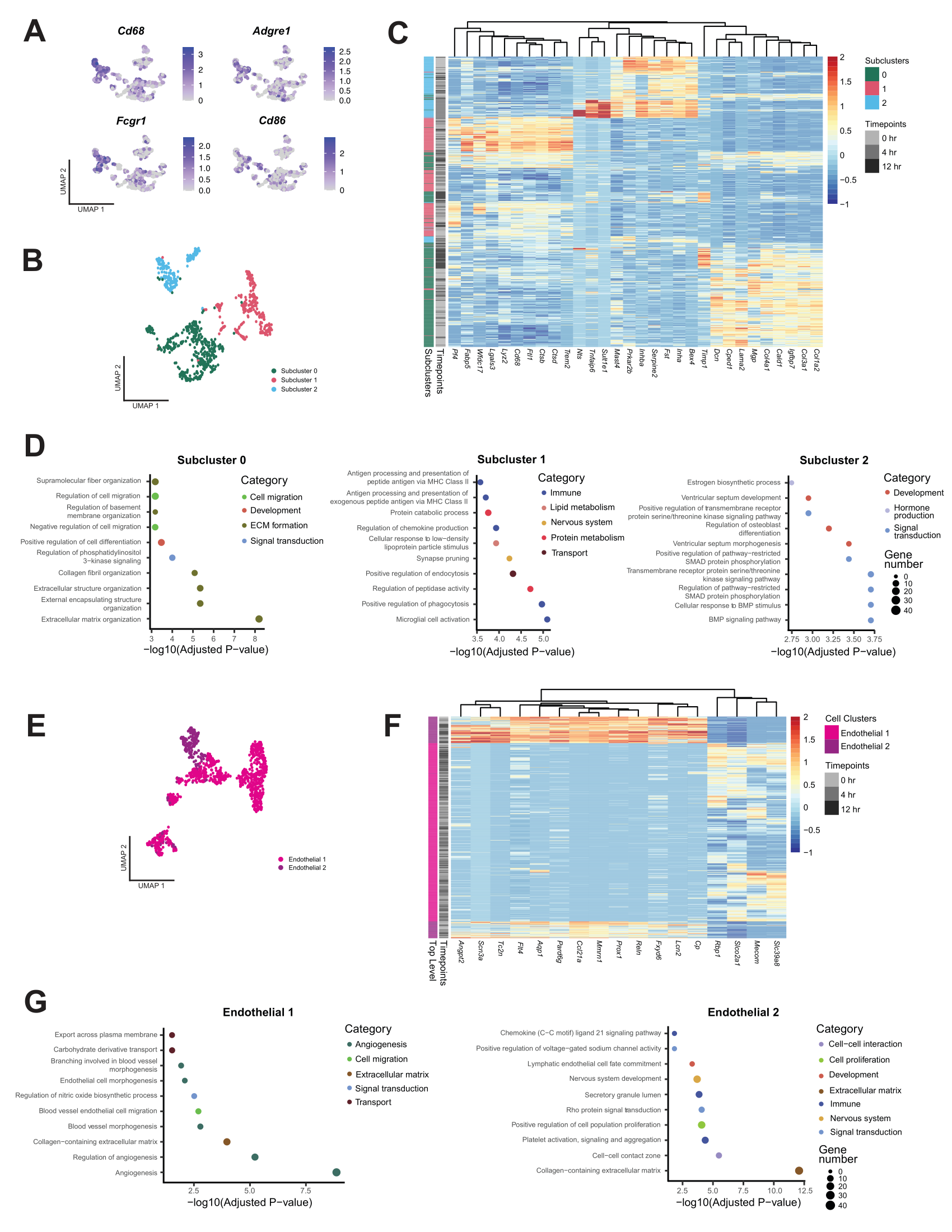

Supplement: S7 Fig — (A) Feature plots show expression of macrophage markers (Cd68, Adgre1, Fcgr1, Cd86) within the myeloid cluster from scRNA-seq. (B) UMAP shows the clustering of myeloid cells at the subcluster level from scRNA-seq. (C) Heatmap depicts differential gene expression in myeloid subclusters (Subcluster 0, 1, and 2) from scRNA-seq. (D) Dot plots show top processes upregulated in Myeloid Subcluster 0 (left), Subcluster 1 (middle), and Subcluster 2 (right). (E) UMAP shows the clustering of endothelial cells at the top-level from scRNA-seq. (F) Heatmap depicts differential gene expression in Endothelial 1 and Endothelial 2 clusters from scRNA-seq. (G) Dot plots show top processes upregulated in Endothelial 1 (left) and Endothelial 2 (right) clusters. The data underlying this figure is available at the Gene Expression Omnibus (GEO) under accession number GSE294534. (TIFF) [file pbio.3003193.s007.tiff]

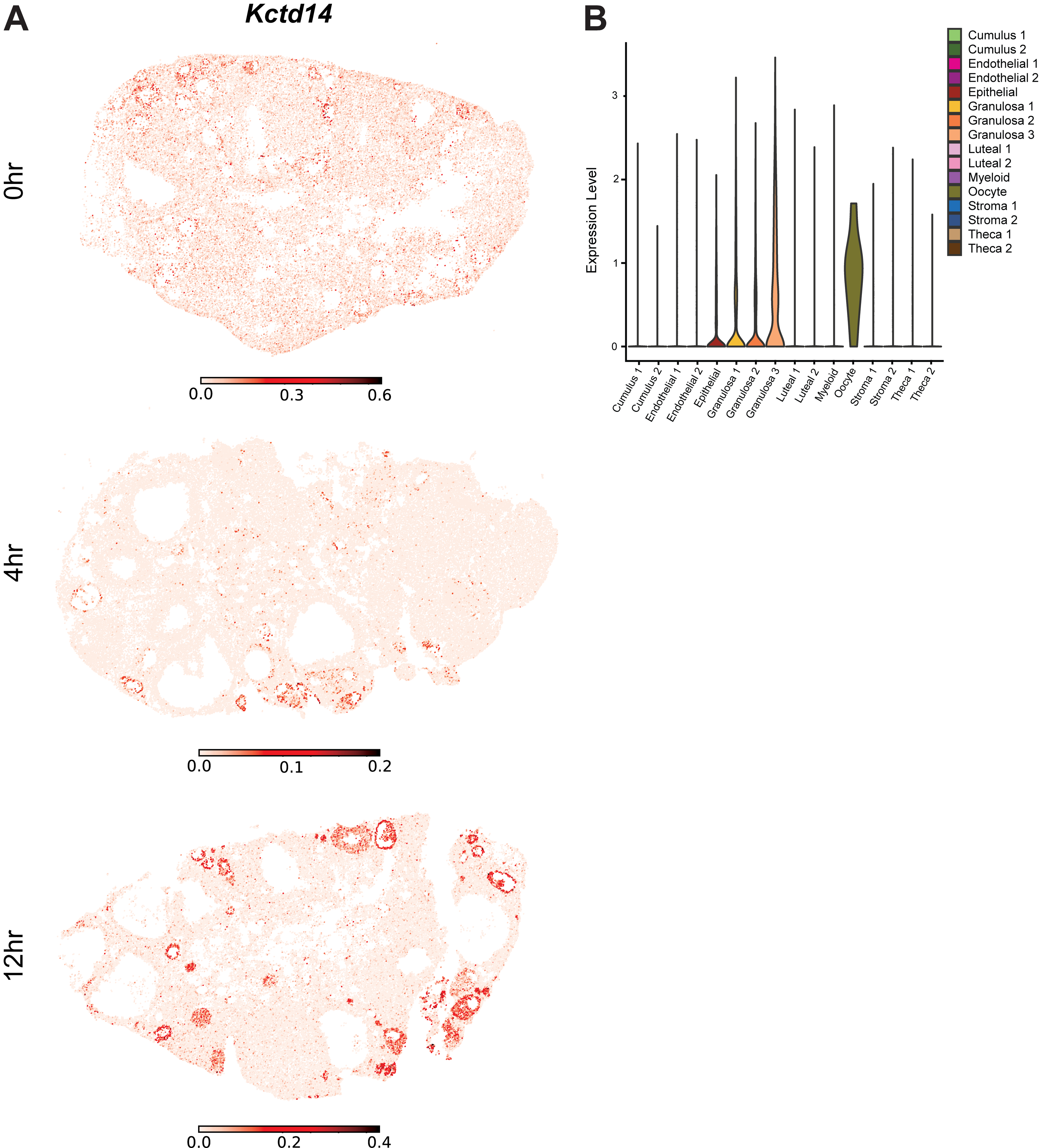

Supplement: S10 Fig — (A) Inferred expression of Kctd14 from the integration of scRNA-seq and iST data shows localization to preantral follicles. (B) Violin plot shows Kctd14 expression in granulosa cell clusters, including Granulosa 3. The data underlying this figure is available at the Gene Expression Omnibus (GEO) under accession number GSE294534. (TIFF) [file pbio.3003193.s010.tiff]
