# Supplementary material for: Single-cell and spatiotemporal profile of ovulation in the mouse ovary
Source: PLoS Biol. 2025 Jun 24;23(6):e3003193. doi: 10.1371/journal.pbio.3003193 (PMC12186953; doi:10.1371/journal.pbio.3003193)
Supplement: Attachment — Submitted filename: Reviewers_Response_(PBIOLOGY-D-24-02509R1)_auresp_3.docx [file pbio.3003193.s019.docx]

We thank the Senior Editor, Academic Editor, the editorial team, and reviewers for their comments and feedback which has served to significantly strengthen our study.

**Senior Editor’s comments:**

As you will see below, the reviewers comment that the study offers a useful and comprehensive dataset. However they have also provided a number of suggestions to strengthen the study further and to make some of the findings clearer and we think these points should be carefully addressed before publication. As a last note, we would not require that you substantially expand the study with new analyses, as suggested by Reviewer 2. Reviewer 2 has suggested that you develop the study further by analyzing the data more deeply and/or by showing how the current study and approach goes beyond previous characterizations. While we think this certainly would be interesting, after discussion with the Academic Editor and the other reviewers, we think the study and its results already fits within the scope of our 'Methods and Resources' article type, and we would not require that it be substantially expanded.

**Response:** We appreciate the comments provided by the Senior Editor. We have taken the considerations provided by the reviewers, the editorial team, the Academic Editor, and the Senior Editor and have incorporated them into our revised manuscript. We have addressed the comments of Reviewer 2 where possible while keeping within the scope of a ‘Methods and Resources’ article. Many of these suggestions correspond to ongoing areas of investigation in our laboratories.

**Reviewer: 1**

**Comment #1:** Throughout the manuscript it would be advantageous for each major cell type (stromal, theca, luteal etc.) to include a subset of DEG markers defining each cluster (say top5-10 by p-value or LogFC in a heatmap, violinplot, dotplot or equivalent) in the corresponding figure or as tables. It would make the manuscript a more straightforward resource when quickly comparing gene lists. It would also make it immediately more intuitive what the genes chosen to be validated correspond to.

**Response 1:** DEGs for each major cell type have been provided as a supplementary table (**Supplemental File 1)**. However, we agree with the reviewer’s recommendation and have instead included this information in the main manuscript as **Table 1** so that it is more accessible.

**Comment #2:** In Fig3- the nomenclature of theca1 and theca 2 as early and late, and subcluster naming of 0h-theca1 and 0h-theca2 could lead to confusion, perhaps rename the latter. Do they correspond to immature and steroidogenic theca? There is no exploration of the DEG between 0h-theca1 and 0h-theca2. The clustering in B makes it difficult to identify the salient markers for each cluster type.

**Response 2:** We appreciate this reviewer’s comments about the nomenclature used to describe our theca clusters. As is typical in single-cell studies, we have identified top-level clusters that correspond to major cell types as shown in **Figure 1**, and we then performed sub-clustering analysis on each cell type in further figures. Theca 1 and Theca 2 represent top-level cell types, and sub-clustering revealed further diversity in these cells once they were subsetted from the dataset. We agree that this is confusing, so we have clarified this in our text (**Lines 211-213**), reproduced here for ease:

*“To further investigate the time-dependent changes in gene expression for each cell type, we performed sub-clustering analyses where possible, followed by pathway analyses on cluster-defining marker genes and differential gene expression analysis (DEA) between early and late cell subclusters for time-varying cell types.”*

We have additionally adjusted the labeling of the theca subclusters at 0h as Theca_0hA and Theca_0hB and this is reflected in the manuscript text (**Lines 273 and 275**) and **Figure 3**.

Next, to determine if any of these subclusters are immature or steroidogenic, we performed module scoring with our dataset against genes that have been previously identified as having a role in marking theca cells and in steroidogenesis (Morris *et al.,* 2022) (**Reviewer Response Figure 1**). Here, you can see that only Theca_12 scored slightly higher for this module, indicating that this cluster may possibly contain steroidogenic theca cells. Scoring for early Theca markers suggests that Theca_00A (Theca_0hA), 00B (0h-Theca_0hB), and Theca_04 may have earlier signatures compared to Theca_12. However, given this data, we are hesitant to make strong claims about these subclusters and have instead provided detailed DEGs for these in **Supplemental File 3**.

**A.	B.**

**Reviewer Response Figure 1. Theca subcluster module scoring.** Violin plots depicting module scoring for (A) the top 10 steroidogenic theca markers and (B) the top 10 early theca markers from Morris et al., 2022.

**Comment #3:** Line 287: Do you have evidence the cluster includes both granulosa and theca cells differentiating into luteal cells rather than just granulosa cells?

**Response 3:** We acknowledge that theca cells present at earlier ovulation timepoints may differentiate and contribute to luteal cells. To further investigate whether the identified cluster includes both granulosa and theca cells differentiating into luteal cells, we performed a pseudotime trajectory analysis on different cell groupings, including (1) luteal and theca cells, (2) granulosa and theca cells, and (3) luteal, granulosa, and theca cells. The premise is that pseudotime analyses would reveal clear differentiation trajectories to luteal cells and granulosa cells. However, in all cases analyzed, the cells did not form clear trajectory-like transcriptomic patterns, nor did they align well with the expected developmental timeline (**Reviewer Response Figure 2**). This suggests that the differentiation process is not strongly represented in the dataset or that the transition signatures are not distinct enough to resolve clear trajectories.

**A.	B.**

**C.	D.**

F)

**E.	F.**

**Reviewer Response Figure 2.** **Pseudotime trajectory analysis of theca, granulosa, and luteal cell clusters.** Plots depicting the pseudotime trajectory of (A-B) granulosa and luteal cells, (C-D) luteal and theca cells, and (E-F) granulosa, luteal, and theca cells. Plots A, C, and E are grouped by cell type and plots B, D, and F are grouped by pseudotime. Pseudotime analysis was performed using SCORPIUS (Cannoodt et al., 2016).

**Comment 4:** Line 349: Cumulus 1 and cumulus 2 do not include any cells from the 0h condition. It is likely that the "0h" cluster was too different to be picked up as a distinct cluster within the granulosa cell supercluster. Cumulus cells are transcriptionally almost identical to preantral granulosa cells, whereas antral mural gc will be closer to the luteinizing mural. Your MERFISH likely includes examples of this if looking at markers like Kctd14.

**Response 4:** We agree that there are no distinct cumulus cell clusters identified at 0h. We might expect this cell to be too rare and thus not detectable in our single-cell RNA-seq dataset or our spatial dataset. Additionally, we find that *Kctd14*, which was previously identified to be enriched in preantral granulosa cells, is also expressed in preantral follicles in our spatial dataset (**Reviewer Response Figure 3A**). In our single-cell dataset, *Kctd14* is enriched primarily in the Granulosa 3 cluster (**Reviewer Response Figure 3B**), suggesting that our Granulosa 3 cluster may be used to distinguish preantral follicles and may include 0h cumulus cells.

We have acknowledged this excellent point in our discussion (**Lines 608-611**):

*Although we did not identify a specific cumulus cell cluster at 0h, we find that Kctd14, which was previously identified to be enriched in a cluster of preantral granulosa/cumulus cells (Morris et al., 2022), is also expressed in preantral follicles in our datasets, particularly in our Granulosa 3 cluster (data not shown). Thus, 0h cumulus cells may be incorporated alongside granulosa cells within the Granulosa 3 cluster.*

**A.**

**B.**

**Reviewer Response Figure 3. Spatial and single cell plots of *Kctd14* across ovulation timepoints.** (A) Expression plots demonstrating predicted *Kctd14* expression/localization identified in mouse ovaries at 0h, 4h, and 12h post-hCG. (B) Violin plot showing *Kctd14* expression for each major ovarian cell cluster identified via single cell sequencing.

**Comment 5:** Fig 5 - This figure doesn't have the temporal expression violin plots you have in the previous figures, presumably because the cumulus 0h wasn't identified. Still, the message would be easier to understand with the corresponding expression data for the targets chosen for validation across those two timepoints.

**Response 5:** The reviewer is correct that we did not show the temporal expression plots in **Figure 5** because we did not capture a 0h cluster that exclusively contained cumulus cells. Moreover, the tissue section utilized for MERFISH contained very few complete COCs for analysis, given that only one tissue section was used per ovary. Therefore, we used histologic sections of ovaries generated across the same ovulation time course as the ovaries collected for single-cell and spatial transcriptomics. In these samples, adjacent sections are only 5 µm apart, allowing us to capture entire COCs across sections so that we could select the best section containing complete COC structures for target validation. Based on the reviewer’s comment, we have clarified the decision to use histological sections for this figure in **Lines 368-371**.

**Comment 6:** There is a conspicuous absence of discussion of the myeloid and endothelial clusters despite their relatively large representation in the dataset. Given their potential role in ovulation, it would be of interest to have at least some superficial analysis of DEG across timepoints. Even fewer than expected differences would be interesting.

**Response 6:** We agree that both the myeloid and endothelial clusters play a crucial role during ovulation and therefore to address this point, we conducted further analyses of these clusters which we have now included as Supplemental Figures in our revised manuscript.

For the myeloid cells, we found that this cell cluster primarily consists of macrophages as indicated by the expression of known markers (*Cd68*, *Adgre1*, *Fcgr1*, *Cd86*) (**Reviewer Response Figure 4A**). Additionally, we conducted a subcluster analysis and identified three subclusters (Subcluster 0, Subcluster 1, Subcluster 2) (**Reviewer Response Figure 4B**). Although these subclusters did not differ in their temporal profile, they exhibited very different gene expression profiles when comparing their DEGs. Compared to the other subclusters, Myeloid Subcluster 1 is enriched for conventional myeloid markers (*Lyz2*, *C1qa*, *C1qb*, *C1bc*, *Apoe*, *Tyrobp*) as well as pathways involved in antigen presentation, cytokine production, and phagocytosis **(Reviewer Response Figure 4D-E, 4G)**. During ovulation, macrophages infiltrate the ovary and secrete cytokines, proteases, and vascular growth factors to induce inflammation and support follicle rupture (Wu et al., 2004; Fedorcsák et al., 2007; Duffy et al., 2019). In addition, they have been shown to recognize and phagocytose atretic granulosa cells, suggesting a role in clearing up local cell debris in ovulating follicles (Wu et al., 2004).

However, the top DEGs for Myeloid Subcluster 0 contained genes that were related to adhesion, and pathway analysis showed enrichment for pathways involved in ECM organization, degradation, and signaling **(Reviewer Response Figure 4D-F)**. These findings are consistent with the known roles of macrophages and monocytes in producing proteases (Werb *et al.,* 1980; Owen and Campbell, 1999), which may contribute to follicle wall and ovarian surface remodeling during ovulation. Macrophages also secrete interleukins such as IL-1A and IL-1B, which induce the expression of proteinases and gelatinases (Gérard *et al.,* 2004; Dang *et al.,* 2017) that may also be involved in follicle wall or ovarian surface epithelium degradation. Myeloid Subcluster 2 showed high expression of genes involved in hormone signaling (*Inhba*, *Inhbb*, *Inha*) and biosynthesis (*Hsd17b1*, *Hsd3b1*, *Cyp19a1*) in the ovary and exhibited upregulation of pathways related to ovulation and hormone production (**Reviewer Response Figure 4D-E, 4H**).

It is possible that the different transcriptional identities of the Myeloid subclusters is secondary to myeloid cells phagocytosing other cell types, and thus exhibiting gene profiles similar to other ovarian cells. However, an alternative explanation is that myeloid cells may have inherent plasticity, which allows them to adopt some of the characteristics of cells they are adjacent to or interact with. This possibility is further supported by our spatial dataset (**Figure 1F, Supplemental Fig 4**) which shows that myeloid cells are present across the ovary (stroma, follicles, corpus lutea), as well as our cell-cell interaction analysis (**Figure 6A, B**) which indicates that myeloid cells receive most of their incoming signals from stroma cells. It has also been previously shown that the cumulus and the ovarian stroma acquire immune and inflammatory signatures during ovulation (Hernadez-Gonzalez *et al*., 2006; Duffy *et al*., 2019). Therefore, it is possible that there is bidirectional communication between myeloid and ovarian cell types that also contribute to myeloid cells acquiring other transcriptomic signatures. One limitation of this subcluster analysis is that we cannot determine the spatial localization of these Myeloid subclusters. Because differential expression analysis was performed directly between subclusters, shared myeloid markers amongst subclusters will be absent in the top DEG list for some subclusters (in this case, Myeloid Subclusters 2 and 3). As a result, these subclusters will include genes expressed in other top-level clusters, masking the specific expression of Myeloid subclusters 2 and 3. However, these analyses provide a better understanding of how myeloid cells may transcriptionally change throughout ovulation. These details have been added in a separate paragraph within the Results (**Lines 415-438**) and Discussion (**Lines 654-665)** as well as now presented as **Supplemental Fig 7** and **Supplemental File 4.**

**Myeloid Subcluster Analysis**

**A.	B.**

**C.**

**D.** *Top 20 DEGs in each subcluster*	**E.**

| **Cluster 0** | **Cluster 1** | **Cluster 2** |
| --- | --- | --- |
| Col1a2 | Lgals3 | Inhba |
| Col3a1 | Ctsd | Sult1e1 |
| Dcn | Fabp5 | Inha |
| Mgp | Lyz2 | Bex4 |
| Timp1 | Ctsb | Nts |
| Igfbp7 | Wfdc17 | Tnfaip6 |
| Lama2 | Ftl1 | Fst |
| Cped1 | Trem2 | Mast4 |
| Cald1 | Cd68 | Prkar2b |
| Col4a1 | Pf4 | Serpine2 |
| Slit3 | Psap | Adgrv1 |
| Cyp11a1 | C1qb | Btc |
| Tshz2 | Tyrobp | Gja1 |
| Dlc1 | Ctss | Bmpr1b |
| Pbx1 | Tmsb4x | Nap1l5 |
| Cacnb2 | Cyba | Ano4 |
| Col1a1 | Fcer1g | Nr5a2 |
| Rbms3 | Apoe | Fshr |
| Pdzrn3 | C1qc | Fam13a |
| Tpm1 | Plin2 | Robo2 |

F**.** *Myeloid Subcluster 0*

**G.** *Myeloid Subcluster 1*

*H***.** *Myeloid Subcluster 2*

**Reviewer Response Figure 4: Subclustering analysis of myeloid cluster.** (A) Feature plots show the expression of macrophage markers (*Cd68*, *Adgre1*, *Fcgr1*, *Cd86*) within the myeloid cluster from scRNA-seq. (B) UMAP shows the clustering of myeloid cells at the subcluster level from scRNA-seq. (C) Bar plot shows the percent of cells in myeloid subclusters expressed at each timepoint. (D) Top 20 differentially expressed genes for each myeloid subcluster. (E) Heatmap depicts differential gene expression in myeloid subclusters (Subcluster 0, 1, and 2) from scRNA-seq. (F) Dot plots show top processes upregulated in Myeloid Subcluster 0. (G) Dot plots show top processes upregulated in Myeloid Subcluster 1. (H) Dot plots show top processes upregulated in Myeloid Subcluster 2.

For the Endothelial clusters, we conducted DEG and pathway analysis to determine the identity of each cluster. We found that Endothelial 1 is enriched for genes involved in hematopoiesis (*Pcp4l1*, *Mecom*, *Kitl*) and angiogenesis, including *Flt1* (also known as *Vegfr1*), *Cd34*, and *Nrp1* (**Reviewer Response Figure 5B-C**). Indeed, pathway analysis of Endothelial 1 shows enrichment in endothelial cell morphogenesis, blood vessel morphogenesis/migration, and angiogenesis (**Reviewer Response Figure 5D**). The importance of ovarian blood vasculature for follicle development and survival, as well as oocyte competence, has been extensively demonstrated in the literature (Kosaka *et al.,* 2007; Reynolds *et al.,* 1992; Greenwald, 1989). In particular, blood vessels transport key nutrients, signals, and metabolic precursors to the follicle and deliver waste products away from the follicle (Brown and Russell, 2014). During ovulation, immune cell recruitment to the follicle is mediated by blood vasculature (Duffy *et al.,* 2019). Follicular fluid originates from blood within the capillaries of the theca externa, and accumulates in the antrum of the follicle in a process fundamental to ovulation (Rodgers and Irving-Rodgers, 2010). Angiogenesis is also one of the primary processes occurring during corpus luteum development, and these newly formed blood vessels are important for progesterone efflux to support a potential pregnancy (Brown and Russell, 2014). In contrast to Endothelial 1, Endothelial 2 is enriched for marker genes for lymphatic endothelial cells (*Ccl21a, Lyve1, Prox1, Flt4*) and for pathways involved in lymphatic endothelial cell fate commitment and platelet activation (**Reviewer Response Figure 5B-C, 5E**). In general, lymphatic vessels are critical for both interstitial fluid homeostasis as well as transporting proteins and immune cells (Guo *et al.,* 2024). In the ovary, the lymphatics have known roles in luteolysis (Abe *et al.,* 2014), and may also transport hormones to and from the bloodstream. During ovulation specifically, lymphatic vessels likely contribute to immune cell influx (Duffy *et al.,* 2019; Guo *et al.,* 2024). Considering their role in extravascular fluid maintenance, it is also possible that the lymphatics contribute to fluid accumulation within antral follicles (Brown *et al.,* 2010). Although these two endothelial clusters do not exhibit temporal differences, their transcriptomic profile indicates that they may facilitate unique roles within the ovary throughout ovulation. These additional analyses and interpretations have been included in **Lines 440-468** and **Supplemental Fig 7.**

**Endothelial Cluster Analysis**

**A.**	**B.** *Top 20 DEGs in each subcluster*

| **Endothelial 1** | **Endothelial 2** |
| --- | --- |
| Flt1 | Ccl21a |
| Rbp1 | Mmrn1 |
| Ctla2a | Reln |
| Sparc | Lyve1 |
| Slco2a1 | Fgl2 |
| Igfbp7 | Prox1 |
| Cd34 | Aqp1 |
| Ly6a | Lcn2 |
| Ly6c1 | Cp |
| Esam | Flt4 |
| Insr | Scn3a |
| Slc39a8 | Pard6g |
| Fam167b | Angpt2 |
| Nrp1 | Fxyd6 |
| Mecom | Tc2n |
| Ptprg | Gpm6a |
| Nostrin | Galnt18 |
| Adamts9 | Rnase4 |
| Pcp4l1 | Pcsk6 |
| Kitl | Stab1 |

**C.**

**D.** *Endothelial 1*

**E.** *Endothelial 2*

**Reviewer Response Figure 5:** Endothelial cells exhibit multiple cell populations during ovulation. (A) UMAP shows the clustering of endothelial cells at the top-level from scRNA-seq. (B) Top 20 differentially expressed genes for each endothelial cluster. (C) Heatmap depicts differential gene expression in Endothelial 1 and Endothelial 2 clusters from scRNA-seq. (D) Dot plots show top processes upregulated in Endothelial 1 cluster. (E) Dot plots show top processes upregulated in Endothelial 2 cluster.

**Comment 7:** Could you include a list of probes used for MERFISH.

**Response 7:** We have now included a new supplemental table that includes the list of probes used for MERFISH (**Supplementary File 2**).

**Comment 8:** In fig1- I greatly appreciate the consistent color scheme for the cell clusters, but panel 1C could still use headings at the top.

**Response 8:** We have now added headings to Figure 1C.

**Reviewer: 2**

**Comments to the Author:** The authors comprehensively analyzed how gene expression patterns change in various ovarian cells during ovulation by single-cell RNAseq and MERSCOR techniques. Although there have been reports of single cell RNAseq in all ovarian cells during the estrus cycle, there have been no reports of detailed time-dependent analysis of the ovulation process, so this database is considered to be useful for clarifying ovulation phenomena from multiple perspectives. In addition, the value of this database is further enhanced by the fact that the MERSCOR technique simultaneously shows where the cell groups of each cluster analyzed by single cell RNAseq are localized.

**Response:** We thank the reviewer for highlighting the strengths of our study.

**Comment 1:** On the other hand, the results of this analysis are almost identical to those of granulosa cells and cumulus cells that have been performed by microarray analysis and RNAseq for more than 20 years. This indicates the appropriateness of the experimental method of this study by combining single-cell RNAseq and MERSCOR technology. However, it can be said that new academic knowledge is scarce.

**Response 1:** We thank the reviewer for their acknowledgment of the appropriateness of using single-cell RNAseq and MERSCOPE technology for this study, and that our results validate what has been previously published in granulosa and cumulus cells using other techniques such as quantitative real-time PCR (qRT-PCR), suppression subtractive hybridization, microarrays, and RNA-seq analysis (Eppig *et al.*, 2005; Hernandez-Gonzalez *et al.*, 2006; Diaz *et al.*, 2007; Borgbo *et al.*, 2013; Yerushalmi *et al.*, 2014; Wigglesworth *et al*., 2015; Morris *et al.*, 2022). As such, the validation of known marker genes and pathways provides us and the field with a robust resource to explore novel genes and pathways within our integrated dataset. Whereas other transcriptomic technologies require the selection of candidate genes *a priori*, single-cell RNA sequencing provides the ability to evaluate over 25,000 genes in an unbiased approach. Indeed, we reported on several highly enriched genes that were not previously identified in cumulus cells, including *Zfp804a*, *Tspo*, *S100a6*, and *Rnf180* (**Figure 5**). Profiling of the entire transcriptome at the single-cell level also allows for unbiased transcriptomic analyses to identify unique cell populations based solely on their gene expression profiles rather than isolation of specific cell types. Notably, our unbiased cluster analysis not only identified cumulus cells separately from mural granulosa cells but also two cumulus clusters with unique temporal and transcriptomic profiles (**Figure 1**). In contrast, previous transcriptomic studies have not separated cumulus from granulosa cells or only found one cumulus cluster across the ovulation time course (Morris *et al.*, 2022; Mantri *et al.*, 2024). Given that follicle development, cumulus expansion, and ovulation rely on continuous crosstalk between ovarian cell types within and outside of the follicle, our technological approach of single-cell transcriptomics (for cell-cell interaction analysis) paired with an integrated spatial dataset is uniquely positioned to comprehensively determine cell-cell communication that is not possible using the other methodologies mentioned. We observed that cumulus and granulosa cells both receive and output signals from other ovarian cell types, including stroma, epithelial, and myeloid (**Figure 6)**. These observations align with our data showing enriched immune and neuronal pathways in the early cumulus (Cumulus 1) cluster (**Figure 5**). Notably, our subcluster analysis on myeloid cells also identified a subpopulation that adopts a cumulus gene expression profile (see **Reviewer 1** **Response #6**). Indeed, we see spatially that myeloid cells are present within preovulatory follicles during ovulation (**Figure 1F, Supplemental Fig 4**), further supporting potential bidirectional crosstalk. Overall, these examples showcase our integrated single-cell and transcriptomic dataset and its advantages in further characterizing the unique but connected role of cumulus cells throughout ovulation. As a resource paper, this dataset provides a platform to further investigate novel genes, pathways, and interactions that may play a crucial role during ovulation.

**Comment 2:** However, unlike the exhaustive detection methods of the past, this research method should be able to dig deeper into the differences between cells within each cluster at each sample time. Based on the diversity and localization analysis within each cluster, it is expected that more detailed analysis will be possible, such as the difference in expression patterns between the cumulus cells in the vicinity of the oocytes and the follicular cavity, and the expression patterns in granulosa cells differ between the follicular membrane side and the follicle cavity side. Unfortunately, this paper only makes a rough classification, such as dividing cumulus cells into different clusters in the early and late ovulation phases.

**Response 2:** We agree with the reviewer that it would be ideal to conduct a more detailed analysis that included differences in expression within a cell type based on proximity to key ovarian structures. However, because the sections used for MERFISH constitute 2D snapshots of the ovary, they lack the resolution necessary to evaluate three-dimensional follicles or COCs. In addition, sections must be chosen before running MERFISH, meaning that sections are not necessarily optimized to capture features that later become of interest after analysis. In our study, these sections only captured a limited number of COCs. To conduct this analysis comprehensively, adjacent sections or 3D imaging would be required. We have added these approaches as future directions to our discussion.

As spatial transcriptomics technologies continue to advance, higher-resolution methods may enable a more refined characterization of cellular heterogeneity within the follicle. Future developments in multi-modal imaging approaches may further enhance our ability to spatially resolve transcriptomic differences at subcellular resolution. These innovations will likely make it possible to explore the cellular microenvironment of ovarian structures in even greater detail.

**Comment 3:** If this paper is to be a database or methodological paper, the differences from past detection methods should be shown by dividing them into advantages and disadvantages. If researchers combine existing transcriptome analysis with IHC, they may be able to obtain similar experimental results. How do we answer this question?

**Response 3:** We appreciate the reviewer’s suggestion and recognize that combining traditional transcriptomic methods with immunohistochemistry (IHC) could provide spatial insights for selected genes. However, such an approach has several key limitations. While IHC can localize proteins within tissues, it is limited by the availability of validated antibodies, the number of proteins that can be simultaneously detected, and the lack of transcript-level resolution. Additionally, traditional transcriptomic methods such as qRT-PCR, northern blots, and microarrays allow for the targeted detection of selected genes but require investigators to predefine their genes-of-interest, making them unsuitable for unbiased discovery. Although early RNA sequencing resolves some of these issues by enabling whole-transcriptome analysis, it still lacks spatial resolution and requires dissociated cells, causing a loss of critical information about the cellular context and microenvironmental influences. These limitations are particularly important in the ovary, where cellular organization and interactions play crucial roles in various biological processes such as follicle development, ovulation, and corpus luteum formation. To address these challenges, spatial transcriptomics combined with single-cell offers a powerful alternative by combining whole-transcriptome analysis with in situ hybridization, enabling the evaluation of cell expression profiles within complex spatial structures. For the ovary, this information is critical given the dynamics of follicle development, ovulation, and corpus luteum formation throughout ovulation. Although spatial transcriptomic technologies are rapidly advancing, one current disadvantage is its lack of single-cell resolution. Depending on the technology, they may also lack transcript coverage (for image-based methods) or sequencing depth (for sequenced-based methods) compared to scRNA-seq. The power of our dataset, which integrates both scRNA-seq and spatial transcriptomics from paired ovaries, allows for the ability to leverage the strength of both techniques to evaluate over 25,000 genes at single-cell resolution and simultaneously explore their spatial expression pattern throughout ovulation. In response to the reviewer’s comment, we have included this section clarifying the advantages and disadvantages across methods in the Discussion section (**Lines 553-565**).

**Comment 4:** In addition, the experimental method and its value as a database have not been improved by the GO analysis and cell-cell interaction analysis conducted in this paper. If a function prediction is to be made, it should be clarified that the prediction is correct and what its significance is. In addition, the reviewer thinks that such results can be obtained by separating each cell and performing RNAseq, and in fact, several such papers have been published.

**Response 4:** Pairing single-cell and spatial transcriptomics, as done in this study, has identified the expression and localization of genes with previously unknown roles in ovulation, and this opens up a multitude of new avenues for research. As a resource paper, our goal is to make these datasets accessible to the broader scientific community to query, increase the extent of validation, and drive our understanding of ovulation forward. We also appreciate the reviewer’s comment regarding separating cells and performing RNAseq. Beyond oocytes, isolating pure populations of cells from the whole ovary has been historically challenging, especially from the complex and heterogeneous stroma. Therefore, it would be difficult to obtain enough populations of cells from the ovary for RNAseq to yield accurate results. Furthermore, transcriptomics on isolated ovarian cell populations would lose the ability to observe spatial information across cell types and structures within the ovary. Instead, our approach of using single-cell transcriptomics has allowed us to sequence all cell populations within the ovary throughout ovulation with high accuracy and reproducibility, and the addition of spatial transcriptomics allowed us to validate our annotation of detected cell populations. We have described the validity and power of this dataset in the Discussion (**Lines** **568-574**).

**Comment 5:** The reviewer thinks that this paper should focus on demonstrating the correctness of the experimental method and its value as a database. Otherwise, the reviewer would have to point out that the academic value and novelty of the analysis results would be limited.

**Response 5:** We have addressed the value of our methods and this resource over existing methods as described in response to Comments 3 and 4, and this information has been included in the Discussion (**Lines 553-565**). The correctness of the experimental method is evidenced by the ability to recapitulate well-established hallmarks of ovulation biology, including cumulus-oocyte-complex expansion**,** steroid hormone production/signaling, and luteinization.

**Reviewer: 3**

**Comments to the Authors:** In this MS, the Authors through scRNA-seq and iST analyses, we have captured major ovarian cell types and their spatial distributions across 3 time points around stimulated ovulation in CD1 mice. Overall, it is a comprehensive study describing the events of ovulation in the mouse and serves as a robust resource for discovering cell states that are unique to ovulation. Furthermore, the focus on the temporal dynamics of ovulation across a short time frame, employing both scRNA-seq and iST is elegant and innovative and the experimental approach of utilizing one ovary from each mouse for scRNA-seq and the contralateral ovary for iST at each of the specific timepoints is elegant and powerful. This study is acceptable after some corrections and clarifications are made. Please find my comments below:

**Response:** We thank the reviewer for their overall positive assessment of our work.

**Comment 1:** Fig 1F - the color chosen for theca cells is difficult to distinguish from other colors. Where are the theca cells in each time point ? Given the importance of theca cells it would be beneficial to either designate a different color to theca cells or add an additional image of the ovary showing the location of only theca, similar to SF4A, but focusing on theca cells.

**Response 1:** We thank the reviewer for the suggestion and have adjusted the colors for theca cells (Theca 1 and Theca 2). This is now reflected in **Figure 1**. Please refer to **Supplemental Fig S4A** for the spatial images of a theca-enriched marker (Hao2). Although we were able to resolve canonical theca localization around follicles with known theca markers, we also observed some non-specific signals with these markers due to their overlap with the transcriptional profile of stroma cells.

**Comment 2:** Only 16 oocytes were identified and sequenced (Fig. 1C). Do the authors know what caused such low yield of oocytes? In the methods section the authors mentioned that they removed cells that had >20% mitochondrial DNA. It is known that oocytes carry a significantly greater number of mitochondria. How did the authors made sure that they did not exclude oocytes by filtering cells with high mitochondria content.

**Response 2:** The low yield of oocytes is primarily due to the exclusion of mature oocytes since they are too large to pass through the filter used to create a single-cell suspension (Eppig and O’Brian, 1996; Winkler *et al.*, 2024). Oocytes from all follicle stages are also highly sensitive to the tissue dissociation procedure, and their recovery tends to be low (Han et al., 2020; Morris et al., 2022; Wei et al., 2022). Furthermore, oocytes are not ideal candidates for 10x sequencing methods/droplet-based approaches as they tend not to load well into droplets (Machlin and Shikanov, 2022).

To ensure that oocytes were not excluded based on mitochondrial content, we compared the percent of mitochondrial DNA in oocytes pre and post-filtering by mitochondrial content (**Reviewer Response Figure 6**). The percent of mitochondrial DNA pre and post-filtering is similar, showing that mitochondrial DNA content in all oocytes was less than our threshold of 20%, and thus all oocytes were retained through filtering.

In other single-cell transcriptomic datasets of the ovary, similar cutoffs (e.g. 15% mitochondrial DNA) were used and also yielded low oocyte numbers (n=22 in Morris *et al*., 2020). One recently published study increased the threshold for mitochondrial DNA to 25% to ensure the inclusion of oocytes specifically (Isola *et al*., 2024). However, we chose not to increase our threshold for mitochondrial DNA beyond 20% to avoid the inclusion of low-quality cells from other ovarian cell types. Due to the limitations described here, this manuscript is not equipped to be a resource for oocyte biology but instead can be utilized to evaluate the expression profiles of all other major ovarian cell types throughout ovulation. In response to the reviewer’s comment, we have clarified this limitation further in **Lines 691-693**.

**Reviewer Response Figure 6. Mitochondrial content (%) in oocytes pre- and post-filtering.**

**Comment 3:** For the explanation of why oocytes were filtered out provided in the discussion (**Lines 494-495**) "It is likely that the majority of oocytes were filtered out during single-cell preparation suspension, and immature oocytes may have passed through the filter due to their smaller size [118]." do the authors claim that the oocytes were smaller than the somatic cells? What other cells were filtered out in that case? I think an explanation in depth, including sizes that are retained, would sharpen the explanation.

**Response 3:** Mouse oocytes are known to increase in size throughout oogenesis but range from ~20 µm (in primordial follicles) to ~70 µm at terminal size (Eppig & O’Brien, 1996). Given that the filter used to generate a single cell suspension was 30 µm in diameter, we have likely captured some small oocytes from primordial and primary follicles. There is also precedence for this in the literature: in a recently published study that also performed single-cell sequencing of the mouse ovary, the authors note that oocytes were excluded altogether during straining of the single-cell suspension (Winkler *et al.*, 2024). Although selecting a filter with a larger diameter would incorporate more oocytes into the suspension, it would also increase the number of doublets (e.g., two cells attached to each other) that could artificially alter the observed transcriptomic profiles across cell types. Although there are many primordial follicles containing immature oocytes in the mouse ovary, the enzymatic and mechanical digestion techniques used to dissociate ovarian tissue for preparation of single-cell suspensions are harsh on primordial follicles, which may have further reduced the number of oocytes sequenced beyond the limitation of the filter size. Based on the reviewer’s recommendation, these considerations are further explained in the Discussion (**Lines 579-588**).

**Comment 4:** Fig. 1C would it be possible to add the names of the cell types on top of the bar? The colors are quite close, and it would make reading the heat map easier (very minor).

**Response 4:** We have now added cell types on top of the bar for Figure 1C.

**Comment 5:** From the description it was not clear how the authors reconciled the differences between the ovaries at the 12h time point in their transcriptomics analysis. Which ovaries (with what phenotype) did they analyze? How do the authors explain such striking differences? It is critical to analyze additional ovaries to determine whether it was a technical mistake, or mice can have two different phenotypes. Was this phenomenon specific to the mouse where both ovaries looked the same or in each mouse one ovary was lagging?

**Response 5:** We selected the 12 h post-hCG timepoint because it specifically captures a highly dynamic period when follicle rupture is in progress. We have confirmed this in a detailed ovulatory timecourse performed in our laboratory as part of another study in which ovaries were harvested at 0h, 11h, 12h, or 13h post-hCG, and the number of COCs present in the ovary and ampulla of the oviduct at each timepoint were quantified (**Reviewer Response Figure 7**). At 11h post-hCG, ovulation has not occurred yet and COCs are found only in the ovary (with the exception of the ovary/oviduct pair depicted in purple, which has already begun ovulation, highlighting the biological variation between mice). By 12h post-hCG, ~50% of the COCs had been ovulated into the oviduct, and by 13 h post-hCG, the majority of COCs had been ovulated. Due to inherent biological variability, some mice may have ovulated more COCs at 12h post-hCG than others, leading to differences in the number of large antral follicles still containing COCs at this timepoint, which is evident both histologically (**S1C Fig**) and in the ovaries used for spatial analysis (**S4B Fig**) in this study. We did not compare phenotypes across both ovaries from the same mouse, as one ovary from each mouse was utilized for spatial transcriptomics, and the contralateral ovary was dissected and dissociated for single-cell RNA sequencing. However, comparing the extent of ovulation between ovaries from the same mouse could be an interesting area for future investigation.

**Reviewer Response Figure 7.** Graph showing the number of COCs present in the ovary or ovulated into the oviduct at 0h, 11h, 12h, or 13h post injection of human chorionic gonadotropin to induce ovulation. Each color represents a different mouse within a single timepoint and is used to show the number of COCs in the ovary versus oviduct for that mouse.

**Comment 6:** With regards to stroma cell analysis did the authors identify any spatial differences between stroma 1 and stroma 2? Were these two cell types localized differently, or the changes only happened transcriptionally?

**Response 6**: Using our integrated dataset, we plotted the top markers for Stroma 1 and Stroma 2 identified by scRNA-seq and onto the MERFISH sections at each timepoint. Based on this analysis, the differences between Stroma 1 and 2 appear to be primarily transcriptomic in nature as we did not observe any major spatial differences (**Figure 2, Supplemental Fig 4A**).

**Comment 7:** I am not sure why it wasn't included (technical limitation?), but in general, because of the unique iST, higher resolution images zoomed in and spatially mapping the important discoveries in this manuscript related to the early and late stages of theca cells and temporal differences in granulosa and luteinized cells it would significantly elevate the depth and contribution of this dataset to the scientific community. However, if it is a technical limitation it should be mentioned in the paper.

**Response 7:** We thank the reviewer for the suggestions and have generated zoomed-in spatial images to evaluate the spatial and temporal differences of the theca, granulosa, and luteal clusters during ovulation. Using theca marker genes highlighted in **Figure 3**, we showed that marker genes for early theca cells (*Cyp17a1* - Theca 1) are localized around preantral and antral follicles at 0h post-hCG. Marker genes for late theca cells (*Col4a1* - Theca 2) also show localization around antral follicles at 12h post-hCG but are less specific, given their additional expression in the stroma. For granulosa cells, marker genes for early granulosa cells (*Inhbb* - Granulosa 1) are present primarily within antral follicles and correspond with mural granulosa cells at 0h post-hCG. In contrast, marker genes for late granulosa cells (*Amh* - Granulosa 3) are present primarily in preantral follicles at 12h post-hCG. We also compared the temporal and spatial changes of theca and granulosa cells with luteal cells using marker genes highlighted in **Figure 4**. Specifically, we found that early luteal cells (*Gm2a* - Luteal 1) are primarily found in the corpus lutea from previous estrous cycles at 0hr. In contrast, marker genes for late luteal cells (*Fndc3b* - Luteal 2) are primarily localized to mural granulosa cells of antral follicles at 12hr post-hCG (corresponding with mural luteal cells). We have now included these zoomed-in images in **Supplemental Fig 6** and included descriptions of the colocalization data in **Lines 322-332.**

**A.	B.**

**C.	D.**

**Supplemental Figure 6. Co-localization of theca or granulosa and luteal cells during early and late timepoints of ovulation.** Expression plot shows colocalization of (A) *Cyp17a1* (Blue: Theca 1) and *Gm2a* (Red: Luteal 1) at 0hr, (B) *Col4a1* (Blue: Theca 2) and *Fndc3b* (Red: Luteal 2) at 12hr, (C) *Inhbb* (Blue: Granulosa 1) and *Gm2a* (Red: Luteal 1) at 0hr, and (D) *Amh* (Blue: Granulosa 3) and *Fndc3b* (Red: Luteal 2) at 12hr. Antral follicles are highlighted by dashed circles.

**Comment 8:** A similar comment to #7 also goes towards the two types of endothelial cells and their importance during peri- and postovulatory remodeling and the comment about stroma cells and angiogenesis markers and cell-cell interactions of Ang-Egfr.

**Response 8:** We generated zoomed-in spatial images to evaluate the localization of the endothelial clusters compared to the stromal clusters during early and late ovulation. Using a marker gene for Endothelial 1 (*Flt1*) cells, we observed their co-localization with the ovarian stroma (*Dcn* - Stroma 1) adjacent to antral follicles at 0h post-hCG. At 12h post-hCG, Endothelial 1 cells remain distributed within the stroma (with more puncta present) but are also within corpus lutea. In response to **Reviewer 1 Comment/Response #6**, we identified Endothelial 1 as an endothelial cell type associated with angiogenesis and blood vessel morphogenesis. As such, the observed spatial differences during late ovulation align with increased ovarian vasculature within the ovarian stroma and corpus lutea. Similarly, Endothelial 2 (marked by *Gng11*) cells show spatial and temporal expression patterns similar to Endothelial 1. We identified Endothelial 2 as potentially lymphatic endothelial cells and thus may contribute to the development of the lymphatic vasculature network during ovulation. We have now included these zoomed-in images into **Supplemental Fig 8** and included descriptions of the colocalization data in **Lines 448-450, 454-456, 462-464.** In response to the reviewer’s suggestion, we also investigated the spatial distribution of Ang-Egfr, which was an enriched interaction between theca and luteal cells identified at 12hr post-hCG (**Figure 6**). While *Ang* and *Egfr* are present in theca cells and luteal cells, respectively, their spatial profile suggests that they are also highly expressed in other areas such as the stroma (both) and corpus luteum (Ang) (**Reviewer Response Figure 8**).

**A.	B.**

**C.	D.**

**Supplemental Figure 8. Co-localization of endothelial and stromal cells during early and late timepoints of ovulation.** Expression plot shows colocalization of (A) *Flt1* (Blue: Endothelial 1) and *Dcn* (Red: Stroma 1) at 0hr, (B) *Flt1* (Blue: Endothelial 1) and *Pdgfra* (Red: Stroma 2) at 12hr, (C) *Gng11* (Blue: Endothelial 2) and *Dcn* (Red: Stroma 1) at 0hr, and (D) *Gng11* (Blue: Endothelial 2) and *Pdgfra* (Red: Stroma 2) at 12hr. Antral follicles are highlighted by dashed circles.

**Reviewer Response Figure 8. Co-localization of Ang (theca) and Egfr (luteal) at 12hr post-hCG.** Antral follicles are highlighted by dashed circles.

**References**

Abe, H., Al-zi'abi, M. O., Sekizawa, F., Acosta, T. J., Skarzynski, D. J., & Okuda, K. (2014). Lymphatic involvement in the disappearance of steroidogenic cells from the corpus luteum during luteolysis. *PloS One*, *9*(2), e88953. https://doi.org/10.1371/journal.pone.0088953

Borgbo, T., Povlsen, B. B., Andersen, C. Y., Borup, R., Humaidan, P., & Grøndahl, M. L. (2013). Comparison of gene expression profiles in granulosa and cumulus cells after ovulation induction with either human chorionic gonadotropin or a gonadotropin-releasing hormone agonist trigger. *Fertility and Sterility*, *100*(4), 994–1001. https://doi.org/10.1016/j.fertnstert.2013.05.038

Brown, H. M., Robker, R. L., & Russell, D. L. (2010). Development and hormonal regulation of the ovarian lymphatic vasculature. *Endocrinology*, *151*(11), 5446–5455. https://doi.org/10.1210/en.2010-0629

Brown, H. M., & Russell, D. L. (2014). Blood and lymphatic vasculature in the ovary: development, function and disease. *Human Reproduction Update*, *20*(1), 29–39. https://doi.org/10.1093/humupd/dmt049

Cannoodt R., Saelens W., Sichien D., Tavernier S., Janssens S., Guilliams M., Lambrecht B., De Preter K., Saeys Y. (2016). SCORPIUS improves trajectory inference and identifies novel modules in dendritic cell development. bioRxiv 079509; doi: https://doi.org/10.1101/079509

Dang, X., Zhu, Q., He, Y., Wang, Y., Lu, Y., Li, X., Qi, J., Wu, H., & Sun, Y. (2017). IL-1β Upregulates StAR and Progesterone Production Through the ERK1/2- and p38-Mediated CREB Signaling Pathways in Human Granulosa-Lutein Cells. *Endocrinology*, *158*(10), 3281–3291. https://doi.org/10.1210/en.2017-00029

Diaz, F. J., Wigglesworth, K., & Eppig, J. J. (2007). Oocytes are required for the preantral granulosa cell to cumulus cell transition in mice. *Developmental Biology*, *305*(1), 300–311. https://doi.org/10.1016/j.ydbio.2007.02.019

Duffy, D. M., Ko, C., Jo, M., Brannstrom, M., & Curry, T. E. (2019). Ovulation: Parallels With Inflammatory Processes. *Endocrine Reviews*, *40*(2), 369–416. https://doi.org/10.1210/er.2018-00075

Eppig, J. J., & O'Brien, M. J. (1996). Development in vitro of mouse oocytes from primordial follicles. *Biology of Reproduction*, *54*(1), 197–207. https://doi.org/10.1095/biolreprod54.1.197

Eppig, J. J., Pendola, F. L., Wigglesworth, K., & Pendola, J. K. (2005). Mouse oocytes regulate metabolic cooperativity between granulosa cells and oocytes: amino acid transport. *Biology of Reproduction*, *73*(2), 351–357. https://doi.org/10.1095/biolreprod.105.041798

Fedorcsák, P., Ráki, M., & Storeng, R. (2007). Characterization and depletion of leukocytes from cells isolated from the pre-ovulatory ovarian follicle. *Human Reproduction*, *22*(4), 989–994. https://doi.org/10.1093/humrep/del491

Gérard, N., Caillaud, M., Martoriati, A., Goudet, G., & Lalmanach, A. C. (2004). The interleukin-1 system and female reproduction. *The Journal of Endocrinology*, *180*(2), 203–212. https://doi.org/10.1677/joe.0.1800203

Guo, Y., Xue, L., Tang, W., Xiong, J., Chen, D., Dai, Y., Wu, C., Wei, S., Dai, J., Wu, M., & Wang, S. (2024). Ovarian microenvironment: challenges and opportunities in protecting against chemotherapy-associated ovarian damage. *Human Reproduction Update*, *30*(5), 614–647. https://doi.org/10.1093/humupd/dmae020

Han, X., Wang, R., Zhou, Y., Fei, L., Sun, H., Lai, S., Saadatpour, A., Zhou, Z., Chen, H., Ye, F., Huang, D., Xu, Y., Huang, W., Jiang, M., Jiang, X., Mao, J., Chen, Y., Lu, C., Xie, J., Fang, Q., … Guo, G. (2018). Mapping the Mouse Cell Atlas by Microwell-Seq. *Cell*, *172*(5), 1091–1107.e17. https://doi.org/10.1016/j.cell.2018.02.001

Hernandez-Gonzalez, I., Gonzalez-Robayna, I., Shimada, M., Wayne, C. M., Ochsner, S. A., White, L., & Richards, J. S. (2006). Gene expression profiles of cumulus cell oocyte complexes during ovulation reveal cumulus cells express neuronal and immune-related genes: does this expand their role in the ovulation process?. *Molecular Endocrinology*, *20*(6), 1300–1321. https://doi.org/10.1210/me.2005-0420

Isola, J. V. V., Ocañas, S. R., Hubbart, C. R., Ko, S., Mondal, S. A., Hense, J. D., Carter, H. N. C., Schneider, A., Kovats, S., Alberola-Ila, J., Freeman, W. M., & Stout, M. B. (2024). A single-cell atlas of the aging mouse ovary. *Nature Aging*, *4*(1), 145–162. https://doi.org/10.1038/s43587-023-00552-5

Kosaka, N., Sudo, N., Miyamoto, A., & Shimizu, T. (2007). Vascular endothelial growth factor (VEGF) suppresses ovarian granulosa cell apoptosis in vitro. *Biochemical and Biophysical Research Communications*, *363*(3), 733–737. https://doi.org/10.1016/j.bbrc.2007.09.061

Machlin, J. H., & Shikanov, A. (2022). Single-cell RNA-sequencing of retrieved human oocytes and eggs in clinical practice and for human ovarian cell atlasing. *Molecular Reproduction and Development*, *89*(12), 597–607. https://doi.org/10.1002/mrd.23648

Mantri, M., Zhang, H. H., Spanos, E., Ren, Y. A., & De Vlaminck, I. (2024). A spatiotemporal molecular atlas of the ovulating mouse ovary. *Proceedings of the National Academy of Sciences*, *121*(5), e2317418121. https://doi.org/10.1073/pnas.2317418121

Morris, M. E., Meinsohn, M. C., Chauvin, M., Saatcioglu, H. D., Kashiwagi, A., Sicher, N. A., Nguyen, N., Yuan, S., Stavely, R., Hyun, M., Donahoe, P. K., Sabatini, B. L., & Pépin, D. (2022). A single-cell atlas of the cycling murine ovary. *eLife*, *11*, e77239. https://doi.org/10.7554/eLife.77239

Owen, C. A., & Campbell, E. J. (1999). The cell biology of leukocyte-mediated proteolysis. *Journal of Leukocyte Biology*, *65*(2), 137–150. https://doi.org/10.1002/jlb.65.2.137

Reynolds, L. P., Killilea, S. D., & Redmer, D. A. (1992). Angiogenesis in the female reproductive system. *FASEB*, *6*(3), 886–892.

Rodgers, R. J., & Irving-Rodgers, H. F. (2010). Formation of the ovarian follicular antrum and follicular fluid. *Biology of Reproduction*, *82*(6), 1021–1029. https://doi.org/10.1095/biolreprod.109.082941

Wei, Y., Yu, R., Cheng, S., Zhou, P., Mo, S., He, C., Deng, C., Wu, P., Liu, H., & Cao, C. (2022). Single-cell profiling of mouse and primate ovaries identifies high levels of *EGFR* for stromal cells in ovarian aging. *Molecular Therapy. Nucleic acids*, *31*, 1–12. https://doi.org/10.1016/j.omtn.2022.11.020

Werb, Z., Banda, M. J., & Jones, P. A. (1980). Degradation of connective tissue matrices by macrophages. I. Proteolysis of elastin, glycoproteins, and collagen by proteinases isolated from macrophages. *The Journal of experimental medicine*, *152*(5), 1340–1357. https://doi.org/10.1084/jem.152.5.1340

Wigglesworth, K., Lee, K. B., Emori, C., Sugiura, K., & Eppig, J. J. (2015). Transcriptomic diversification of developing cumulus and mural granulosa cells in mouse ovarian follicles. *Biology of Reproduction*, *92*(1), 23. https://doi.org/10.1095/biolreprod.114.121756

Winkler, I., Tolkachov, A., Lammers, F., Lacour, P., Daugelaite, K., Schneider, N., Koch, M. L., Panten, J., Grünschläger, F., Poth, T., Ávila, B. M., Schneider, A., Haas, S., Odom, D. T., & Gonçalves, Â. (2024). The cycling and aging mouse female reproductive tract at single-cell resolution. *Cell*, *187*(4), 981–998.e25. https://doi.org/10.1016/j.cell.2024.01.021

Wu, R., Van der Hoek, K. H., Ryan, N. K., Norman, R. J., & Robker, R. L. (2004). Macrophage contributions to ovarian function. *Human Reproduction Update*, *10*(2), 119–133. https://doi.org/10.1093/humupd/dmh011

Yerushalmi, G. M., Salmon-Divon, M., Yung, Y., Maman, E., Kedem, A., Ophir, L., Elemento, O., Coticchio, G., Dal Canto, M., Mignini Renzinu, M., Fadini, R., & Hourvitz, A. (2014). Characterization of the human cumulus cell transcriptome during final follicular maturation and ovulation. *Molecular Human Reproduction*, *20*(8), 719–735. https://doi.org/10.1093/molehr/gau031
